# Cultivated Tomato (*Solanum lycopersicum* L.) Suffered a Severe Cytoplasmic Bottleneck during Domestication: Implications from Chloroplast Genomes

**DOI:** 10.3390/plants9111443

**Published:** 2020-10-26

**Authors:** Rachele Tamburino, Lorenza Sannino, Donata Cafasso, Concita Cantarella, Luigi Orrù, Teodoro Cardi, Salvatore Cozzolino, Nunzio D’Agostino, Nunzia Scotti

**Affiliations:** 1 CNR-IBBR, National Research Council of Italy, Institute of Biosciences and BioResources, Via Università 133, 80055 Portici (NA), Italy; rachele.tamburino@ibbr.cnr.it (R.T.); lorenza.sannino@ibbr.cnr.it (L.S.); 2 Department of Biology, University of Naples Federico II, Via Cinthia, 80126 Naples, Italy; cafasso@unina.it (D.C.); cozzolin@unina.it (S.C.); 3 CREA Research Centre for Vegetable and Ornamental Crops, Via dei Cavalleggeri 25, 84098 Pontecagnano Faiano (SA), Italy; concita.cantarella@gmail.com (C.C.); teodoro.cardi@crea.gov.it (T.C.); nunzio.dagostino@unina.it (N.D.); 4 CREA Research Centre for Genomics and Bioinformatics, via S. Protaso 302, 29017 Fiorenzuola d’Arda (PC), Italy; luigi.orru@crea.gov.it; 5 Department of Agricultural Sciences, University of Naples Federico II, Via Università 133, 80055 Portici (NA), Italy

**Keywords:** next-generation sequencing, *Solanum*, Italian landraces, plastome, molecular markers, phylogenetic analysis

## Abstract

In various crops, genetic bottlenecks occurring through domestication can limit crop resilience to biotic and abiotic stresses. In the present study, we investigated nucleotide diversity in tomato chloroplast genome through sequencing seven plastomes of cultivated accessions from the Campania region (Southern Italy) and two wild species among the closest (*Solanum pimpinellifolium*) and most distantly related (*S. neorickii*) species to cultivated tomatoes. Comparative analyses among the chloroplast genomes sequenced in this work and those available in GenBank allowed evaluating the variability of plastomes and defining phylogenetic relationships. A dramatic reduction in genetic diversity was detected in cultivated tomatoes, nonetheless, a few *de novo* mutations, which still differentiated the cultivated tomatoes from the closest wild relative *S. pimpinellifolium*, were detected and are potentially utilizable as diagnostic markers. Phylogenetic analyses confirmed that *S. pimpinellifolium* is the closest ancestor of all cultivated tomatoes. Local accessions all clustered together and were strictly related with other cultivated tomatoes (*S. lycopersicum* group). Noteworthy, *S. lycopersicum* var. *cerasiforme* resulted in a mixture of both cultivated and wild tomato genotypes since one of the two analyzed accessions clustered with cultivated tomato, whereas the other with *S. pimpinellifolium*. Overall, our results revealed a very reduced cytoplasmic variability in cultivated tomatoes and suggest the occurrence of a cytoplasmic bottleneck during their domestication.

## 1. Introduction

Domestication of crops was one of the most complex and dynamic processes in plant evolution driven by humans, as it changed the distribution and frequency of plant species on the planet. Crop domestication, through natural or artificial selection, generally results in a reduction of genetic diversity and in the loss of many adaptive traits from wild relatives [1,2]. The analysis of the genetic diversity of wild relatives and cultivated crops provided insight into the geographic and temporal details of domestication, whilst its estimation may provide the basis for developing suitable strategies for crop improvement, conservation and sustainable use [1]. Over past decades, molecular methods have been used to assess genetic diversity and, more recently, high throughput DNA sequencing technologies gave a huge boost to the estimation of genetic and adaptive diversity in crops and model plants [3,4,5,6].

Tomato (*Solanum lycopersicum* L.) is one of the most consumed vegetables in the world and belongs to the Solanaceae family, which includes species with a considerable economic importance (e.g., potato, pepper, eggplant, tobacco, and petunia) [7]. Within this family, *Solanum* is the largest and probably the most economically important genus, including both potatoes and tomatoes [8,9]. The original place of tomato domestication is still debated, however it is very likely that it occurred independently in the Peruvianum and Mexican regions [7]. The cultivated tomato, *S. lycopersicum* is divided into two botanical varieties *S. lycopersicum* var. *cerasiforme* (i.e., cherry tomato) and *S. lycopersicum* var. *lycopersicum*. Cherry tomato is native to the Andean region, but it also occurs in the subtropical areas and grows either as a true wild or cultivated species. For several years, cherry tomato has been considered an evolutionary intermediate between *S. pimpinellifolium*, the closest wild ancestor, and the cultivated *S. lycopersicum*. Recently, genetic studies [10] found cherry tomatoes were a mixture of wild and cultivated forms that likely originated from *S. pimpinellifolium*.

*S. lycopersicum* var. *lycopersicum* derived from cherry tomato through a multiphases process of domestication [11,12]. In particular, Blanca et al. [11] assumed a predomestication in the Andean regions that resulted into a wide morphological diversity of cherry tomatoes; then these genotypes reached Mesoamerica where the true domestication occurred. Here, traditional tomato varieties were developed and spread by Spanish conquistadors in Spain and Italy and, then, in the rest of the World. Since the late 18th century a strong selection activities has taken place in Europe, giving rise to a wide collection of tomato landraces adapted to local cultivation practices and environmental conditions [13,14,15,16]. More recently, these landraces gained increasing attention because of the high quality of fruits, their extended shelf-life and tolerance to environmental stresses [17,18,19]. Accordingly, several studies focused on the genome-wide characterization of the nuclear genetic diversity of various landraces [14,15,16,20,21,22].

Although it has been widely demonstrated the potentiality of cytoplasmic markers to study crop evolution and assess cytoplasmic bottlenecks occurred during the domestication history of several crops (i.e., rice, barley, potato, maize, and wheat) [23,24,25,26,27,28], to date little attention has been given to the analysis of the chloroplast genome in tomato landraces. Furthermore, a deeper knowledge of tomato plastomes would allow a better understanding of nuclear and cytoplasmic genome coevolution, and favor phylogenetic/barcoding studies and novel biotechnological approaches for breeding purposes [29,30,31].

In this work, we reported the complete plastome sequences of seven Italian cultivated tomato accessions grown in the Campania region (Southern Italy) and two wild species, namely *S. pimpinellifolium* and *S. neorickii*. Among Italian tomato accessions we selected the “Corbarino” landrace (processed tomato) characterized by obovoid fruits and moderate shelf-life, and six accessions belonging to the “Vesuviano” landrace (long shelf-life) characterized by hearth-shaped fruits with a pronounced pointed apex. Although they have the same place of origin, analysis based on nuclear single nucleotide polymorphisms (SNPs) showed a different clustering for some of these accessions [22]. The selected wild species are among the phylogenetically closest and most distantly related species to cultivated tomatoes and belong to two different phyletic groups characterized by red/orange- or green-fruited species, respectively. In particular, we aim to estimate the nucleotide diversity of tomato plastomes, inferring phylogenetic relationships, shedding lights on *de novo* mutations likely associated with the domestication and on the potential cytoplasmic bottleneck occurred during such a process.

## 2. Results

### 2.1. Chloroplast Genome Size and Organization

Sequencing of the nine tomato genotypes produced from 6.2 M (ves2001) to over 11.5 M (pol) high quality paired-end reads.

Cultivated tomato accessions, namely cor, pds, pol, ves2001, vfr, and vpz, had exactly the same plastome size (155,435 bp), with the exception of pgl (only one bp shorter), whereas plastome size in wild species was slightly larger in *S. neorickii* 1 (155,515 bp) and smaller in *S. pimpinellifolium* 1 (155,420 bp; Table 1). All genotypes exhibited the typical quadripartite structure of angiosperms plastome, including a pair of inverted repeats (IRs) separated by a large single copy (LSC) and a small single copy (SSC) regions (Table 1).

### 2.2. Genetic Variability and Phylogenetic Analyses

**Figure 1:**
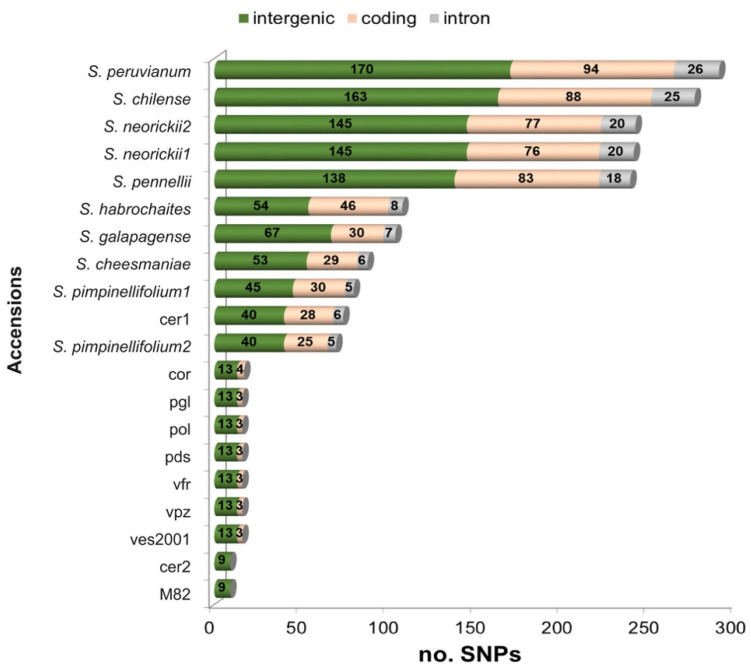
Stacked bar chart showing the distribution of single nucleotide polymorphisms (SNPs) that fall within coding sequences of genes, introns, and intergenic regions of the nine tomato plastomes sequenced in this work and in those of eleven species retrieved from GenBank. The plastome of IPA-6 (AM087200) was used as reference for SNP calling.

Comparative analyses were performed in order to identify patterns of nucleotide variability among the tomato plastomes (the nine genotypes sequenced in this work and the twelve genotypes retrieved from GenBank). An overview of the nucleotide variability was shown in Figure S1. A variable number of SNPs (from a minimum of 9 to a maximum of 290) was observed when cultivated and wild plastomes were compared with the reference genome IPA-6 (Figure 1). Particularly, in cultivated tomatoes the number of SNPs was markedly low (from 9 to 17 SNPs), with the notable exception of cer1 that differed for 74 SNPs from IPA-6, a difference comparable to that of wild *S. pimpinellifolium*. All local accessions showed identical plastome sequences, with the exception of cor that differed for one point mutation in the exon 2 of the *rpoC1* gene and pgl that was one bp shorter.

**Figure 2:**
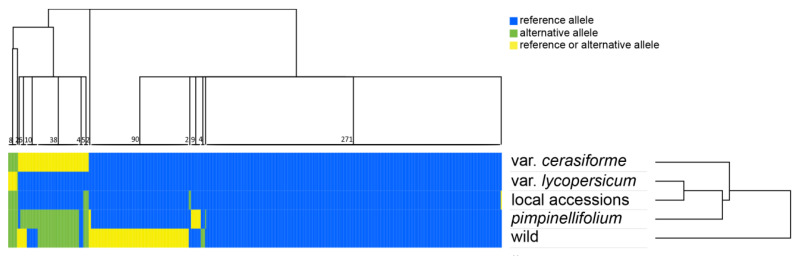
Hierarchical clustered heatmap representing color-coded SNP alleles as scored across 5 different groups of genotypes, i.e., var. *cerasiforme*; var. *lycopersicum*; local accessions; *pimpinellifolium*; wild species (including *S. habrochaites*, *S. cheesmaniae*, and *S. galapagense*). Numbers at the base of the tree indicate the SNP(s) that fall into each group. Blue: reference allele; green: alternative allele; yellow: reference or alternative allele.

Considering the low variability detected, to verify whether the SNPs identified in cultivated genotypes were ancestral or *de novo* mutations likely evolved before or after the domestication process, a comparative analysis was performed on the investigated tomato genotypes clustered into five groups: (1) the *S. lycopersicum* var. *lycopersicum* tomato commercial varieties IPA-6 and M82; (2) the seven local accessions from Campania region; (3) the *S. lycopersicum* var. *cerasiforme* cer1 and cer2; (4) the *S. pimpinellifolium*, *S. pimpinellifolium* 1 and *S. pimpinellifolium* 2, and (5) the wild including *S. habrochaites*, *S. cheesmaniae* and *S. galapagense*, phylogenetically closer to cultivated tomato than other wild species (Figure 2).

Notably, a high number of SNPs (271) was common between all five groups and different from distantly related wild species, thus being ancestral mutations evolved in the phyletic lineages including cultivated tomatoes. Ninety SNPs were common between the cultivated tomatoes (*S. lycopersicum* var. *cerasiforme*, *S. lycopersicum* var. *lycopersicum* and local accessions) and the *S. pimpinellifolium* groups, whereas other wild species showed either the reference or the alternative allele. It is very likely that these latter 90 SNPs have been fixed only in the phyletic lineage of wild *S. pimpinellifolium* and cultivated tomatoes. Only two SNPs distinguished the local accessions from the remaining ones, but 38 SNPs (invariable among the cultivated genotypes) were different between cultivated tomatoes and *S. pimpinellifolium*. Notably, the *S. lycopersicum* var. *cerasiforme* group (cer1 and cer2) showed either the reference or the alternative allele of these 38 SNPs with cer1 sharing the *S. pimpinellifolium* allele, whilst cer2 the cultivated one. This result suggests that these 38 SNPs evolved as *de novo* mutations after the separation of cultivated forms from wild *S. pimpinellifolium* but were already present in the ancestral domesticated gene pool (including the *S. lycopersicum* var. *cerasiforme* group) and only subsequently fixed in the cultivated *S. lycopersicum* var. *lycopersicum* and local accessions groups. The other five SNPs were common between local accessions, *S. pimpinellifolium* and wild groups, while the *S. lycopersicum* var. *lycopersicum* and the *S. lycopersicum* var. *cerasiforme* groups showed, respectively, the reference and either the reference or the alternative allele. By excluding cer1, in cultivated tomatoes seven SNPs (including the latter five and the two exclusive point mutations of local accessions) represent the only differences between plastomes of *S. lycopersicum* var. *lycopersicum* and local accessions.

As expected, wild species showed the highest number of SNPs independently from the phylogenetic distance to the reference genome (Figure 1 and Figure 2) with variation detected even between accessions of the same species: ten different SNPs were found between the two accessions of *S. pimpinellifolium*. By looking at the distribution of SNPs in coding sequences, introns, and intergenic regions, the highest number of SNPs was scored in intergenic regions ranging from 9 to 13 in cultivated tomatoes and cer2, 40 in cer1, and 40-170 in the wild relatives. The same trend was observed for SNP distribution in coding sequences (Figure 1). Particularly, SNPs in wild species ranged from 25 to 94 and were dispersed as 1-2 variations per gene in most genes, whereas among cultivated genotypes up to four SNPs in local accessions were located in *matK*, exon 2 of the *rpoC1*, and *ycf1* coding sequences, one of these being in charge of an amino acid change. In contrast to all other cultivated landraces, once again, cer1 showed the number and distribution of SNPs similar to that found in the wild *S. pimpinellifolium*.

The most variable genes, especially among wild species, were *ndhH* and *ycf1* with 9 and 42 SNPs, respectively (Figure S2 and Figure S3). The mutations observed in the *ndhH* gene were synonymous (i.e., not causing changes in the amino acid sequence), whilst the nucleotide variability observed in *ycf1* was also reflected at the amino acid level. Interestingly, a SNP variation produced an amino acid change between the var. *lycopersicum* and the local accessions (Figure S3).

**Figure 3:**
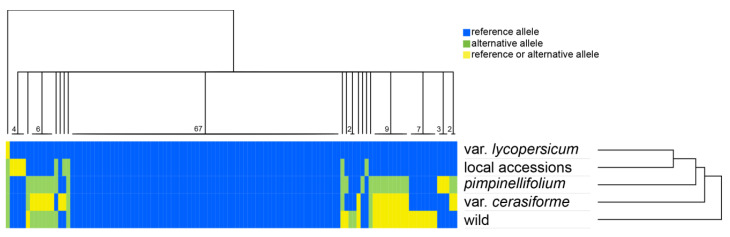
Hierarchical clustered heatmap representing color-coded simple sequence repeat (SSR) alleles as scored across 5 different groups of genotypes, i.e., var. *lycopersicum*; local accessions; *S. pimpinellifolium*; var. *cerasiforme*; wild species (including *S. habrochaites*, S*. cheesmaniae*, and *S. galapagense*). Numbers at the base of the tree indicate the SSR(s) that fall into each group. Blue: reference allele; green: alternative allele; yellow: reference or alternative allele.

One hundred and fourteen simple sequence repeats (SSRs) were identified. The mononucleotide repeat (adenosine or thymine) was the most common type of microsatellite. Only four wild genotypes showed dinucleotide repeats (*S. neorickii* 1 and 2, *S. peruvianum*, and *S. chilense*). As observed for SNPs, clustered heatmap of SSRs across grouped genotypes revealed a very low level of polymorphism (Figure 3).

Sixty-seven SSRs were ancestral (same number of repeat units), being shared by all the analyzed genotypes as compared with the most distantly related wild species not included in the wild group; six SSRs have the same number of repeat units both in *S. lycopersicum* var. *lycopersicum* and local accessions groups, whereas *S. pimpinellifolium* and wild groups displayed a different number of repeat units, and *S. lycopersicum* var. *cerasiforme* both. One SSR displayed 13 repeats shared between *S. lycopersicum* var. *lycopersicum* and local accessions groups (i.e., *atpB-rbcL* intergenic region). An exclusive number of repeat units in the local accessions group was detected in the *ndhC-tRNA-Val* (UAC) intergenic region, while a number of repeat units exclusive of *S. lycopersicum* var. *lycopersicum* group was found in the *psbE-petL* intergenic region (Figure 3, Table S2). Interestingly, one SSR in the *atpH-atpI* intergenic region has the same number of repeat units both in all cultivated genotypes and wild group, while *S. pimpinellifolium* displayed a different number (Table S2). A complete description of SSR variability was shown in Figure S4a. As already observed for SNPs, SSRs were mainly located in intergenic regions (58%) and were mostly included in the LSC (75%; Figure S4b).

Among the in silico identified microsatellites, eight SSR loci with small variation in the number of repeat units were experimentally tested to verify the correct estimation of their length. No variation in the number of repeat units was detected both in silico and in the electrophoresis profiles in a representation of the nine genotypes sequenced in this work and in a large dataset including additional local accessions and processed/fresh market tomatoes (e.g., Acampora, Lucariello, San Marzano, and Sorrento) confirming the absence of SSR variation within and among cultivated tomatoes. A notable exception was the one basis difference found in the microsatellite located in the *ndhF-rpl32* intergenic region that allowed distinguishing local accessions group from other tomato landraces and that was also confirmed by the electrophoresis profiles (data not shown).

**Figure 4:**
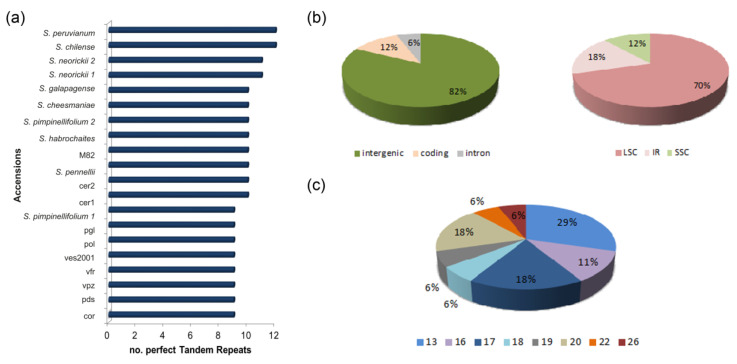
Perfect tandem repeats (TRs) in the nine plastomes sequenced in this work and in the plastome sequence of eleven species available in GenBank. The plastome of IPA-6 (AM087200) was used as reference. (**a**) Bar chart reporting the total number of TRs in each genotype. (**b**) Pie charts describing the percentage of TRs located in the coding sequences of genes, introns, and intergenic regions and in the large single copy (LSC), small single copy (SSC), and inverted repeat b (IR) regions. (**c**) Pie chart describing the percentage of TRs with a specific period size.

Additionally, 17 perfect tandem repeats (TRs) were found, with cultivated species displaying a lower TR number when compared with wild species (Figure 4a). The identified TRs were mainly located in the LSC and intergenic regions (70 and 82%, respectively); two TRs found in all genotypes were in the coding region of the *rps16* and *rps4* genes (Figure 4b). The TR period size ranged from 13 to 26 bp (Figure 4c). TRs confirmed the low variability among the analyzed tomato genotypes. No TR was specific to any cultivated tomato; neither *de novo* TRs could be identified. A TR located in the *tRNA-Gln (UUG)-psbK* intergenic region was the only one to be found variable among species (Table S3). Particularly, local accessions and *S. pimpinellifolium* 1 had one copy, *S. neorickii* 1 and 2 had three copies, while *S. lycopersicum* var. *lycopersicum* (IPA-6 and M82), *S. lycopersicum* var. *cerasiforme* (cer1 and cer2), *S. pimpinellifolium* 2, and the remaining wild species had two copies (Table S3). Interestingly, a *de novo* duplication of four bases motif (ATAA)_2_, exclusive of the local accessions, was scored by MSA (Figure S5).

**Figure 5:**
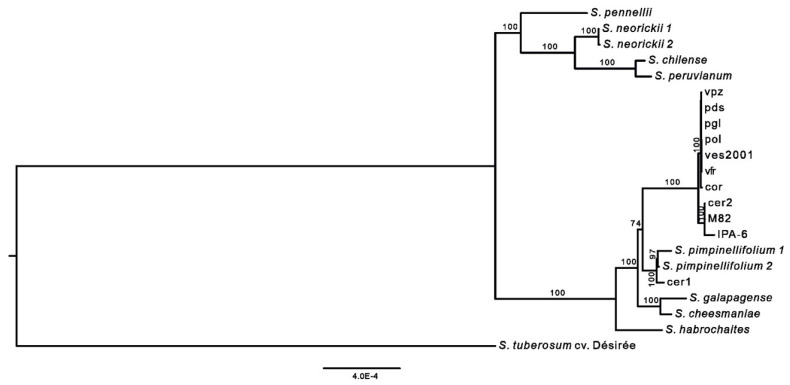
Phylogenetic tree of cultivated and wild tomato genotypes. Phylogram of the best maximum-likelihood (ML) tree on the complete plastome dataset using *Solanum tuberosum* cv. Désirée (DQ386163) as the outgroup. Numbers associated with branches are ML bootstrap support values. Bootstraps higher than 70% are reported on the nodes.

Phylogenetic tree inferred from the complete plastomes of the twenty-one tomato genotypes using the potato chloroplast genome (*S. tuberosum* cv. Désirée, DQ386163) as an outgroup, showed two main clades with strong bootstrap support (100%; Figure 5). One clade included some wild species (*S. pennellii*, *S. neorickii* 1 and 2, *S. peruvianum,* and *S. chilense*) with *S. pennellii* as the basal species. The other clade is further separated into several subclades. In particular, the group that included the seven local accessions from the Campania region was closely related to a cluster populated by other cultivated varieties (IPA-6, M82, and cer2). As expected, all cultivated genotypes were more closely related to the clade comprising the two *S. pimpinellifolium* accessions and cer1. The remaining wild species (*S. galapagense*, *S. cheesmaniae,* and S*. habrochaites*) were in a separate clade. Finally, the phylogenetic analysis confirmed the admixed nature of *S. lycopersicum* var. *cerasiforme* as cer1 was closely related to the wild species (*S. pimpinellifolium* 1 and 2), while cer2 was part of the cultivated genotypes clade (M82 and IPA-6).

## 3. Discussion

Most crops experienced a reduction in genetic diversity (genetic bottleneck) due to the domestication process [32]. Indeed, the development of high yielding crops for food, feed, and other uses required the desirable phenotypes to be selected at the expense of variability present in their wild ancestors (founder effect) [33,34,35]. However, such “uniformity” often resulted in more vulnerable plants that are not able anymore to cope with biotic and abiotic stresses. As a consequence, wild relative species are often exploited as a reservoir of “exotic” alleles to secondarily increase variability in previously selected traits, thus favoring adaptation to changed conditions [34].

Landraces are locally adapted cultivars that are gaining increasing attention considering their typical traits (e.g., high quality of fruits and yield stability in low input agricultural systems) [17,19,36,37,38]. Although it has been widely demonstrated that the chloroplast genome is a valuable resource to study evolution and phylogenetic relationships among species [39,40], the genetic diversity of tomato landraces was largely based on the genome-wide characterization of their nuclear DNA variability [11,12,14,15,16,20,21,22,41]. Further, due the uniparental mode of inheritance, genetic bottleneck in organellar DNA may not necessarily reflect nuclear variability, thus providing additional/complementary information on the domestication process.

Comparative analyses of the nine plastomes sequenced in this work and of twelve plastomes retrieved from GenBank allowed both to evaluate the extent of the genetic bottleneck on the tomato chloroplast genome and define phylogenetic relationships among wild and cultivated accessions. For these aims, SNPs and SSRs were revealed to be more informative than TRs since no specific TR for cultivated tomato genotypes, or *de novo* TRs were identified in our survey.

Very low cpDNA variability was detected in tomato varieties with respect to that observed in wild species, thus indicating the occurrence of a very strong cytoplasmic bottleneck during domestication. The number of SNPs in wild species is 24-fold higher than in cultivated tomatoes (389 polymorphic SNPs out of 454 (86%)), while SSRs were slightly lower (49 polymorphic SSRs out of 114 (43%), 4-fold those observed in tomato varieties). The heterogeneous nature of the *S. lycopersicum* var. *cerasiforme* group is remarkable, namely, the two analyzed accessions showed a different behavior. Collected data and phylogeny clearly highlighted higher variability in cer1 compared with cer2 and suggest that although cer1 belongs to *cerasiforme* group, probably it was not subjected to the domestication process and can be considered as “wild” cultivated accession.

Detected levels of plastome variability are consistent with the extensive genetic erosion of cultivated tomato, especially in the light of the large diversity observed across wild relatives [5]. Similarly, pepper wild species displayed a number of SNP and SSR respectively 8-fold and 3-fold greater than that of cultivated genotypes [42].

Only 16 out of 454 SNPs were found polymorphic among cultivated tomato genotypes (3.5%). Comparable results were found in pepper varieties, where only the 4% of the scored SNP loci were polymorphic in cultivated accessions [42].

Similarly, only 12 out of 114 identified SSRs were polymorphic among cultivated tomato genotypes (11%). Comparable results were reported in cultivated barley showing one polymorphism out of seven analyzed SSRs (14%) [27] and pepper varieties, showing 19 polymorphic SSRs out of 92 (21%) [42]. Contrariwise, 16 out of 17 (94%) SSRs were polymorphic among cultivated bean [43].

As previously argued, genetic bottleneck at the nuclear level may not be reflected at the cytoplasmic level. An extreme cytoplasmic bottleneck has been previously hypothesized in cultivated potato by the analysis of SSR markers but no decreased levels of nuclear SSR diversity were recorded [26,39]. On the contrary, the genetic diversity analysis between American and European collections of common bean highlighted the absence of evident cytoplasmic bottleneck (only 2% loss of cpSSR diversity) [44], and a stronger nuclear bottleneck (30% loss of SSR diversity) [45] likely indicating that the founding common bean populations introduced in Europe were still highly variable in their cytoplasmic DNAs [46].

SNP arrays on some tomato cultivars, partially shared with this work (i.e., M82, cor, pgl, vfr, and ves2001), revealed a reduced nuclear genetic diversity [22].

Concordantly, the cpDNA analyses suggest an extreme low cytoplasmic variability of the founding cultivated tomato population. Indeed, cultivated varieties shared 361 out of 454 SNPs (79%) and 74 out of 114 SSRs (65%) with the ancestor *S. pimpinellifolium* (i.e., same SNP alleles and same SSR haplotypes) and only seven *de novo* SNPs and two *de novo* SSRs were different between *S. lycopersicum* var. *lycopersicum* and local accessions groups. All analyzed local accessions showed identical cpDNA sequences suggesting that these accessions have a unique domestication origin and that their cytoplasm has evolved monophyletically from the founder tomato gene pool, rather than representing an independent introduction. Still, the local accessions have distinctive sequences from the other commercial tomatoes (i.e., *S. lycopersicum* the var. *lycopersicum* group) excluding multiple independent selections of the obovoid fruits (Corbarino) or the hearth-shaped fruits with a pronounced pointed apex (the remaining accessions).

In this work, we also detected plastome variability between wild *S. pimpinellifolium* 1 and 2. These differences could be due to natural variability among accessions and/or possible errors in the sequencing/assembly procedure. The former hypotheses, however, is supported by differences also observed among other related wild species (*S. galapagense*, *S. cheesmaniae,* and *S. habrochaites*). Thus, the significant reduction in cpDNA variability found in the cultivated tomato gene pool can be directly ascribed as a consequence of the domestication process rather than to an already occurred loss of genetic variation in the closest wild relative, *S. pimpinellifolium*. Therefore, the present study suggests that a severe ‘cytoplasmic bottleneck’ occurred during the domestication of tomato, as has been reported in other crops: barley [27], lentil [47], onion [48], and potato [26].

A strict relationship between cultivated tomato varieties and the ancestor *S. pimpinellifolium* was supported by phylogeny. Species belonging to the Lycopersicon group (*S. lycopersicum*, *S. pimpinellifolium*, *S. cheesmaniae*, and *S. galapagense*) [49] form a well-supported clade in agreement with previous phylogenetic studies [5].

In particular, all local accessions clustered together in a subgroup with *S. lycopersicum* var. *lycopersicum* and cer 2. On the contrary, some accessions (i.e., cor, pgl, vfr, and ves2001) were grouped in different clusters based on nuclear SNP genotyping [22]. Noteworthy, cer1 was included in the same group of *S. pimpinellifolium* accessions, thus plastome diversity analysis confirmed the mixed nature of *S. lycopersicum* var. *cerasiforme* as previously observed with the analysis of nuclear variability [10,11,50,51].

The observed low variability of the cultivated tomatoes chloroplast genome can be explained by taking into account both the genetic bottleneck during their domestication and its low mutation rate. Notably, comparison of the plastome sequences of the two modern tomato varieties IPA-6 and Ailsa Craig, the former bred in South America and the latter in Europe, resulted in identical cpDNA sequences, thus demonstrating the stability of plastome in tomato cultivars over a period of at least a few hundred years of separation [52] without the insurgence of any *de novo* mutation. Although low variation is the rule in tomato cpDNA, few plastid regions have been identified that might be exploited as diagnostic markers: two *de novo* SNPs, one SSR and a short sequence duplication (ATAA)_2_ were exclusive of all local accessions, whereas, one SSR was typical of all the var. *lycopersicum* group.

Variability found in all tomato genotypes mainly affected intergenic regions. However, the most variable genes were *ycf1* (showing both synonymous and non-synonymous mutations) and *ndhH*. Both these genes have been proposed as tools to resolve the phylogenetic relationships among closely related genera and species [53,54,55] and at least *ycf1* was found variable even within cultivated plastomes leading to amino acid change (Figure S3).

Overall, our work contributes to the characterization of tomato plastid genomes and their phylogenetic relationships, and especially highlights the severe reduction in variability at plastid DNA as a consequence of the strong genetic bottleneck occurred in the founding population during the domestication process.

## 4. Materials and Methods

### 4.1. Plant Material

Seven Italian cultivated tomato accessions grown in the Campania region (Southern Italy), Corbarino (cor) landrace, and six accessions belonging to the “Vesuviano” landrace, Pollena (pol), PDS (pds), Vesuvio 2001 (ves2001), Vesuviano foglia riccia (vfr), Vesuviano pizzo (vpz), Piennolo giallo (pgl), and two wild tomato species, *S. pimpinellifolium* (LA0722, Peru) and *S. neorickii* (LA2133, Ecuador) were sampled for chloroplast isolation, DNA extraction and sequencing. Drs. M.S. Grillo and S. Grandillo from the CNR, Institute of Bioscience and BioResources, Portici, kindly provided the seeds.

### 4.2. Chloroplast DNA Isolation and Extraction

Plants were kept in the dark for 48 h before harvesting to reduce starch accumulation. Fresh leaves (15–25 g) were collected and used for chloroplast isolation with discontinuous sucrose gradient [56]. Purified chloroplasts were lysed with a detergent and proteins eliminated by proteinase K and phenol/chloroform treatments following the procedure described by [57].

### 4.3. Genome Sequencing, Assembling, and Annotation

DNA samples were sequenced using the GA II Illumina sequencer (2 × 75 paired-end reads with an estimated inset size of 400 bp). Quality check on raw reads was performed using FastQC v.0.11.2 (http://www.bioinformatics.babraham.ac.uk/projects/fastqc/). Then, the fastq_quality_filter utility from the FASTX-toolkit (http://hannonlab.cshl.edu/fastx_toolkit/) was used to remove sequences with a quality score equal or lower than 30 in more than 90% of the read length. Illumina technical sequences were removed by using Trimmomatic v.0.32 [58]. Reference-based assembly was performed using the Columbus module within the Velvet package [59] with a k-mer size of 65. The chloroplast genome sequence of *S. lycopersicum* cv. IPA-6 (AM087200) was used as reference. Contigs were ordered and oriented by using ABACAS [60] for the final assembly. Finally, high quality reads were aligned back onto the assemblies using Bowtie2 [61] with default settings to validate and manually fix errors in the assemblies. Per base genome coverage was computed using the genomecov utility of bedtools version 2.20.1 (Figure S6) [62]. The annotation of chloroplast genomes was performed using GeSeq (https://chlorobox.mpimp-golm.mpg.de/geseq.html). Gene annotations were manually curated using *S. lycopersicum* cv. IPA-6 (AM087200) annotations as reference. Chloroplast genome sequences and annotations produced in this study can be found in GenBank under accession numbers MT811790-MT811798.

### 4.4. Detection and Analysis of Sequence Variations

Single nucleotide variants (SNVs) were identified using the snp-sites tool (https://github.com/sanger-pathogens/snp-sites). Such a tool extracts SNPs from a multiple sequence alignment using the cpDNA of *S. lycopersicum* cv. IPA-6 as reference sequence. SNP annotation was manually curated.

The microsatellite (MISA) identification tool (http://pgrc.ipk-gatersleben.de/misa/) was run to identify microsatellites (SSR) using the unit_size/min_repeats parameters as follows: 1/8, 2/6, 3/5, 4/5, 5/5, 6/5. The Tandem Repeat Finder web tool accessible at https://tandem.bu.edu/trf/trf.basic.submit.html was used to detect perfect tandem repeats with default settings.

In silico identified SSR loci were experimentally tested for variation in the number of repeat units. For this aim, 8 SSR loci were selected from the MISA output by focusing on those with small variation in the number of repeat units to verify the correct estimation of their repeat length. Primers were designed with Primer3 (http://frodo.wi.mit.edu/primer3/). The primer size was set from 18 to 25 bp, the Tm ranged from 51 to 59 °C and the other parameters were set as default (Table S1). For each microsatellite locus, the forward primers were labeled with the different fluorescent dyes 6-FAM, ATTO550, ATTO565, and HEX (Sigma Aldrich, USA). Beside the sequenced local accessions, we applied these primers to 19 additional local genotypes, namely further seven local accessions and twelve processed/fresh market tomatoes.

All PCR amplifications were performed by a Perkin Elmer 9700 thermocycler according to PCR conditions as reported in [63]. The conditions were maintained constant for all loci in order to maximize standardization. Amplified microsatellite products were then genotyped using an Applied Biosystem 3130 automatic sequencer with LIZ (500) as an internal standard and sized with GENEMAPPER software v. 3.7 (Thermo Fisher Scientific-Applera, USA).

Multiple sequence alignments (MSA) were generated using MAFFT version 7 [64] with default settings. Single-nucleotide variants were identified by the snp-sites software [65] using as input the plastomes MSA and the cpDNA of *S. lycopersicum* cv. IPA-6 (AM087200) as reference. To highlight differences among nucleotide sequences of plastomes, MSA were visualized using the NCBI Multiple Sequence Alignment Viewer available at https://www.ncbi.nlm.nih.gov/projects/msaviewer/.

RAxML [66] was used to build a maximum-likelihood (ML) tree with 10,000 rapid bootstrap inferences, a generalized time reversible (GTR) substitution matrix and Gamma model of rate heterogeneity. The plastome of *S. tuberosum* cv. Désirée (DQ386163) was used as the outgroup. The ML tree was visualized with FigTree v.1.4.2 (http://tree.bio.ed.ac.uk/software/figtree/).

In addition to *S. lycopersicum* cv. IPA-6 (AM087200), eleven tomato genotypes available in GenBank: *S. peruvianum* (KP117026), *S. chilense* (KP117021), *S. neorickii* (*S. neorickii* 2, KP117025), *S. pennellii* (HG975452), *S. habrochaites* (KP117023), *S. galapagense* (NC_026878), *S. cheesmaniae* (NC_026876), *S. pimpinellifolium* (*S. pimpinellifolium* 2, KP117027), *S. lycopersicum* (cv M82, HG975525), and *S. lycopersicum* var. *cerasiforme* (cer1, KY887588; and cer2, KY887587) were retrieved for comparative analyses. Heatmaps were generated using Morpheus (https://software.broadinstitute.org/morpheus). Single-linkage hierarchical clustering on both rows and columns was based on the metric “Euclidean distance”.

## Figures and Tables

**Table 1:** Plastome features of the sequenced tomato genotypes.

Code	Species	Cultivar/Accession	Size (Base Pairs)			
			Total	LSC	SSC	IR
Cor	*S. lycopersicum*	Corbarino	155435	85857	18364	25607
Pds	*S. lycopersicum*	PDS	155435	85857	18364	25607
Pgl	*S. lycopersicum*	Piennolo giallo	155434	85857	18363	25607
Pol	*S. lycopersicum*	Pollena	155435	85857	18364	25607
ves2001	*S. lycopersicum*	Vesuvio 2001	155435	85857	18364	25607
Vfr	*S. lycopersicum*	Vesuvio foglia riccia	155435	85857	18364	25607
Vpz	*S. lycopersicum*	Vesuviano pizzo	155435	85857	18364	25607
*S. neorickii* 1	*S. neorickii*	LA2133	155515	85918	18379	25609
*S. pimpinellifolium* 1	*S. pimpinellifolium*	LA0722	155420	85842	18362	25608

## Supplementary Materials

The following are available online at https://www.mdpi.com/2223-7747/9/11/1443/s1, Figure S1: Overview of the nucleotide variability in nine plastomes sequenced in this study and in eleven species available in GenBank. The accession number AM087200 (cv. IPA-6) was used as reference. Red lines represent variable regions, Figure S2: Schematic representation of the nucleotide variability observed in the *ndhH* gene for the plastomes under investigation. Grey bar represents the nucleotide multiple-sequence alignment (MSA) and it is scaled according to the MSA length. Black boxes indicate variable regions in the MSA. Above and below each box, a snapshot of the MSA along with alignment positions is reported, Figure S3: Schematic representation of the amino acid variability observed in the Ycf1 protein for the plastomes under investigation. Grey bar represents the amino acid multiple-sequence alignment (MSA) and it is scaled according to the MSA length. Black boxes indicate variable regions in the MSA. Above and below each box, a snapshot of the MSA along with alignment positions is reported; Figure S4: Simple sequence repeats (SSRs) in nine plastomes sequenced in this study and in eleven species available in GenBank. The plastome of IPA-6 (AM087200) was used as reference a) Heatmap representing differences in SSR size; colors range from red (SSR size larger than the reference) through yellow to blue (SSR size smaller than the reference). Black is for missing SSRs. b) Pie chart describing the percentage of SSRs located in coding sequences of genes, introns, and intergenic regions and in the large single copy (LSC), small single copy (SSC), and inverted repeat b (IR) regions, Figure S5: Multiple-sequence alignment (MSA) of the region harboring the duplicated sequence (ATAA)_2_ scored only in local landraces, Figure S6: Distribution of per-base sequencing depth for each chloroplast genome sequenced in this work. The average coverage per-base is also reported. Table S1: Tomato cpSSR primers developed in this study, Table S2: Simple sequence repeats (SSRs) in the twenty-one tomato chloroplast genomes using IPA-6 (AM087200) as the reference genome. SSRs size, location, and distribution among different regions, namely coding, intron, and intergenic are reported. The unit_size/min_repeats parameters were as follows: 1/8, 2/6, 3/5, 4/5, 5/5, and 6/5. SSRs located in IRa were not counted. SSRs were identified using MISA—microsatellite identification tool (http://pgrc.ipk-gatersleben.de/misa/), Table S3: Tandem repeats (TRs) in the twenty-one tomato chloroplast genomes using IPA-6 (AM087200) as the reference genome. TRs copy number and location are reported. TRs were identified using the tool available at https://tandem.bu.edu/trf/trf.basic.submit.html.
