# Supplementary material for: Cultivated Tomato (Solanum lycopersicum L.) Suffered a Severe Cytoplasmic Bottleneck during Domestication: Implications from Chloroplast Genomes
Source: Plants (Basel). 2020 Oct 26;9(11):1443. doi: 10.3390/plants9111443 (PMC7692331; doi:10.3390/plants9111443)
Supplement: Supplementary file 1 [file plants-09-01443-s001.pdf]

Supplementary materials

Cultivated tomato (*Solanum lycopersicum* L.) suffered a severe cytoplasmic bottleneck during domestication: implications from chloroplast genomes

Rachele Tamburino [1 §], Lorenza Sannino [1§], Donata Cafasso [2], Concita Cantarella [3], Luigi Orrù [4], Teodoro Cardi [3], Salvatore Cozzolino [2], Nunzio D'Agostino [3,5] and Nunzia Scotti [1 *]

[1] CNR-IBBR, National Research Council of Italy, Institute of Biosciences and BioResources, Via Università 133, 80055 Portici (NA), Italy; rachele.tamburino@ibbr.cnr.it; lorenza.sannino@ibbr.cnr.it; nunzia.scotti@ibbr.cnr.it
[2] Department of Biology, University of Naples Federico II, Via Cinthia, 80126 Naples, Italy; cafasso@unina.it; cozzolin@unina.it
[3] CREA Research Centre for Vegetable and Ornamental Crops, Via dei Cavalleggeri 25, 84098 Pontecagnano Faiano (SA), Italy; concita.cantarella@gmail.com; teodoro.cardi@crea.gov.it
[4] CREA Research Centre for Genomics and Bioinformatics, via S. Protaso 302, 29017 Fiorenzuola d'Arda (PC), Italy; luigi.orru@crea.gov.it
[5] Department of Agricultural Sciences, University of Naples Federico II, Via Università 133, 80055 Portici (NA), Italy; nunzio.dagostino@unina.it
§ These authors contributed equally;
* Correspondence: nscotti@unina.it, nunzia.scotti@ibbr.cnr.it; Tel.: +39-081 2539482

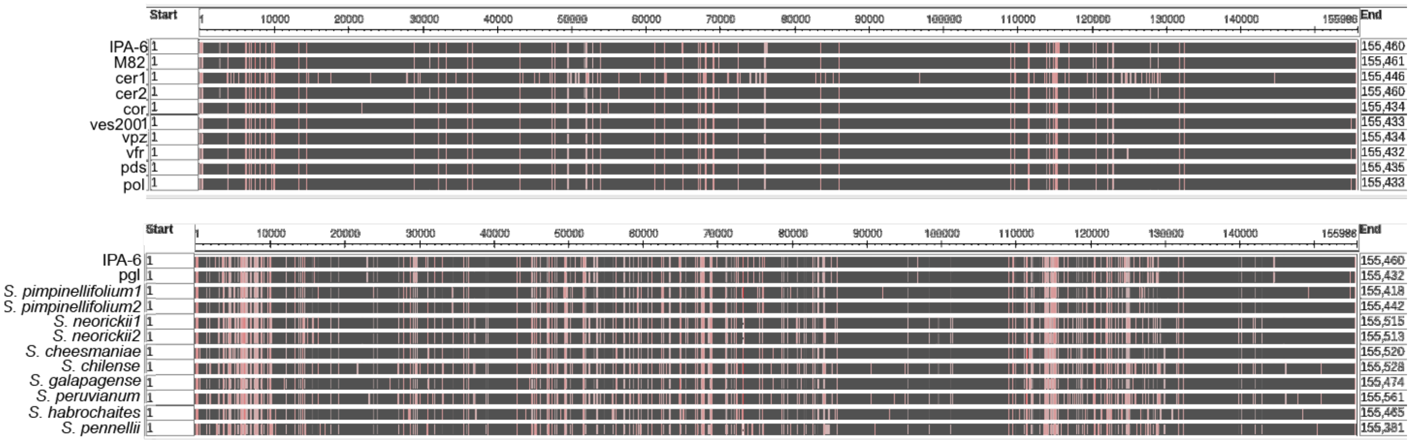

Figure S1. Overview of the nucleotide variability in nine plastomes sequenced in this study and in eleven species available in GenBank. The accession number AM087200 (cv. IPA-6) was used as reference. Red lines represent variable regions.

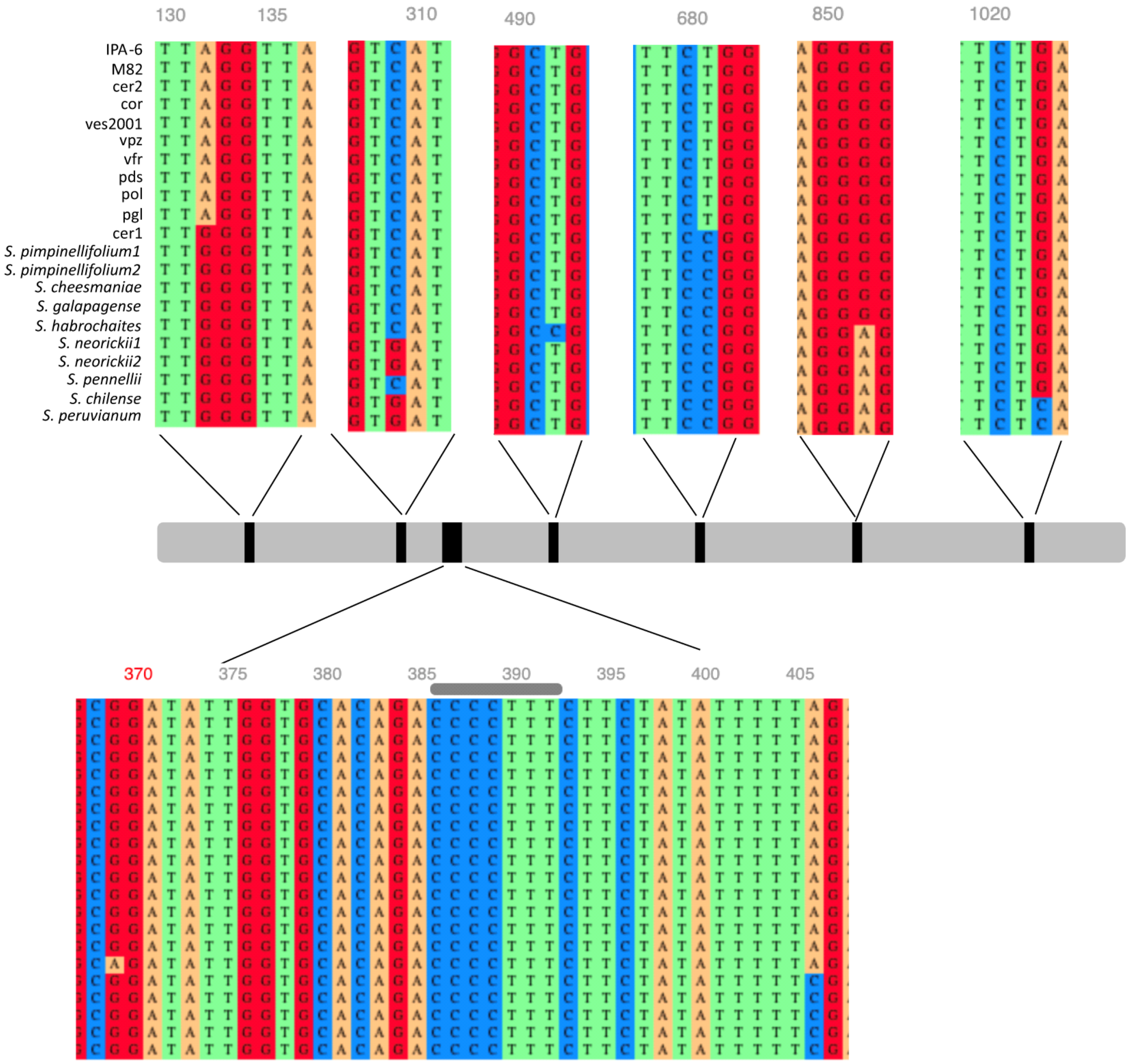

Figure S2. Schematic representation of the nucleotide variability observed in the *ndhH* gene for the plastomes under investigation. Grey bar represents the nucleotide multiple-sequence alignment (MSA) and it is scaled according to the MSA length. Black boxes indicate variable regions in the MSA. Above and below each box, a snapshot of the MSA along with alignment positions is reported.

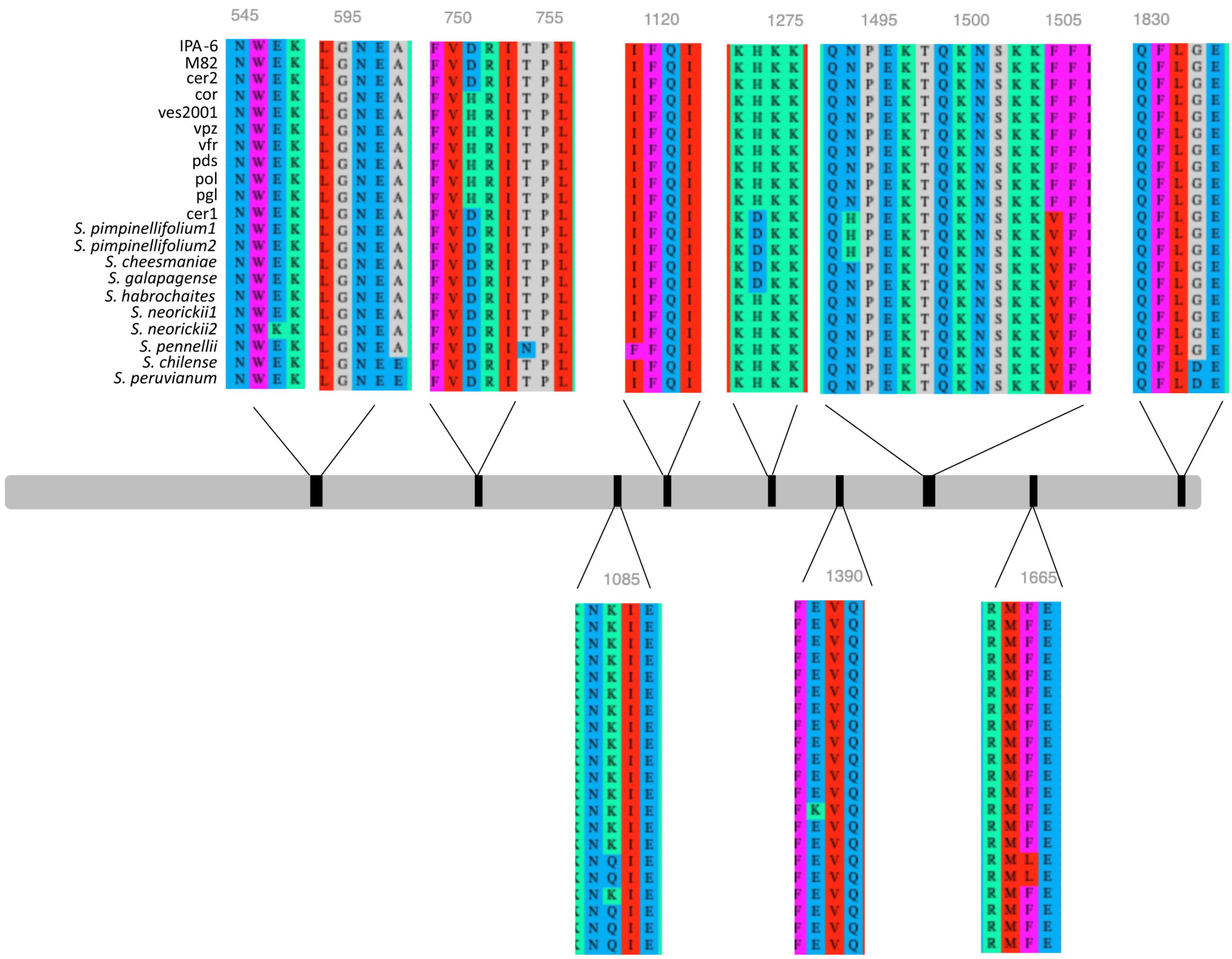

Figure S3. Schematic representation of the amino acid variability observed in the Ycf1 protein for the plastomes under investigation. Grey bar represents the amino acid multiple-sequence alignment (MSA) and it is scaled according to the MSA length. Black boxes indicate variable regions in the MSA. Above and below each box, a snapshot of the MSA along with alignment positions is reported.

a

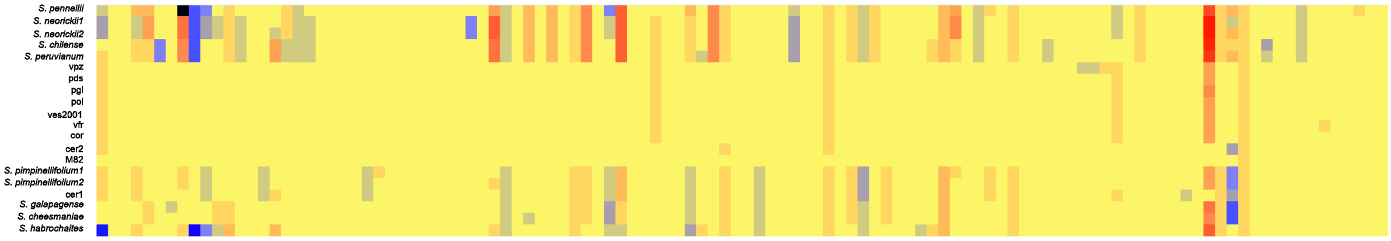

b

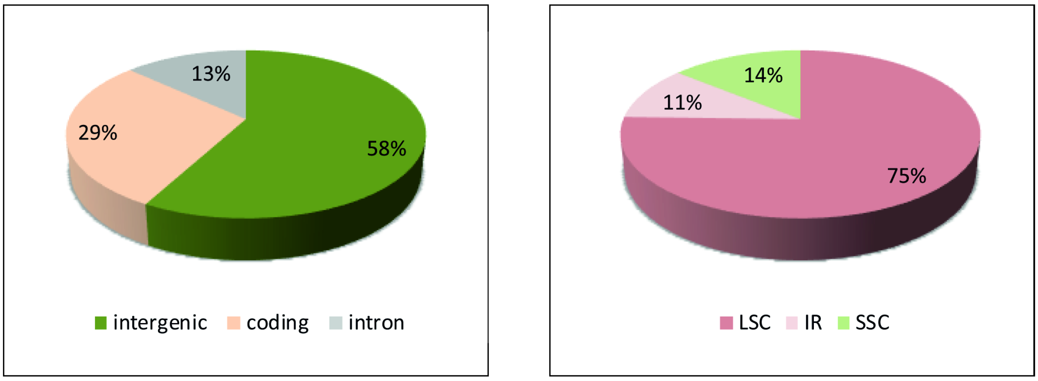

Figure S4. Simple sequence repeats (SSRs) in nine plastomes sequenced in this study and in eleven species available in GenBank. The plastome of IPA-6 (AM087200) was used as reference a) Heatmap representing differences in SSR size; colours range from red (SSR size larger than the reference) through yellow to blue (SSR size smaller than the reference). Black is for missing SSRs. b) Pie chart describing the percentage of SSRs located in coding sequences of genes, introns and intergenic regions as well as in the large single copy (LSC), small single copy (SSC) and inverted repeat b (IR) regions.

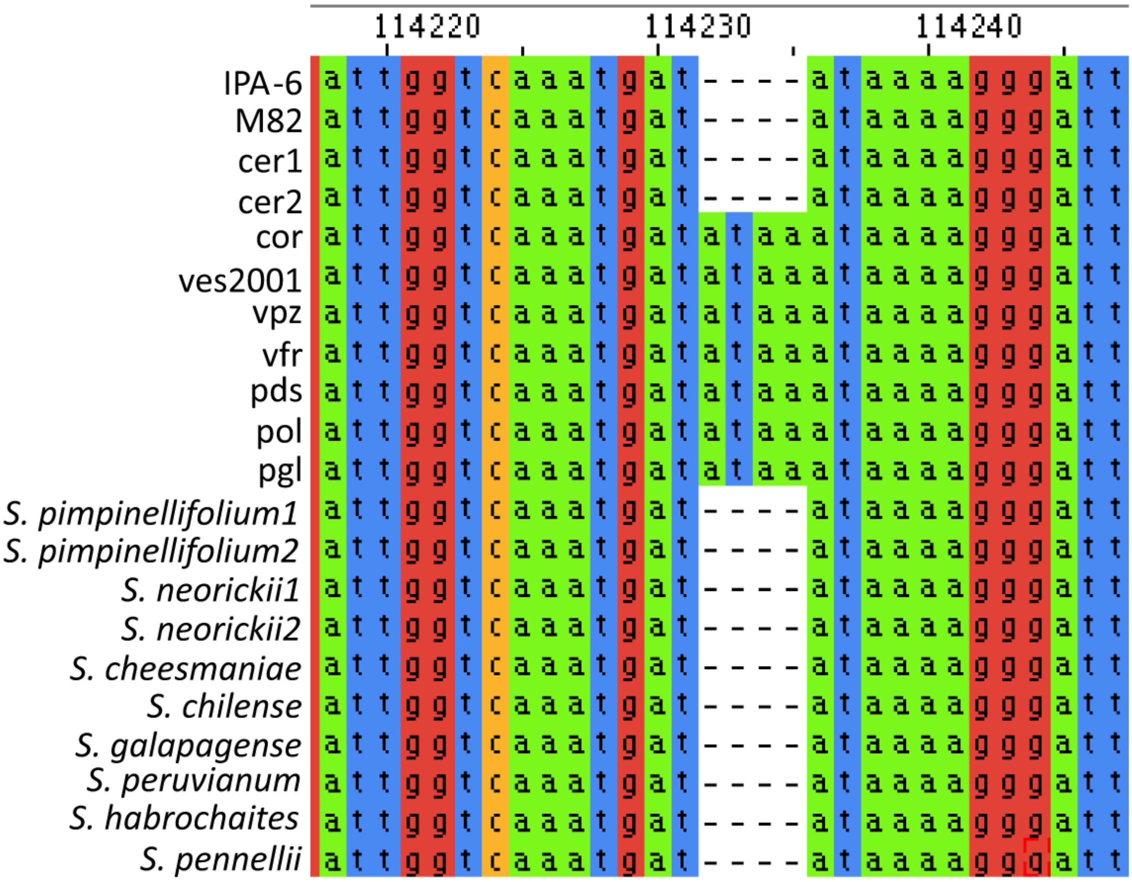

Figure S5. Multiple-sequence alignment (MSA) of the region harbouring the duplicated sequence $(ATAA)_2$ scored only in local landraces.

Corbarino (average read depth/nucleotide: 1072.84)

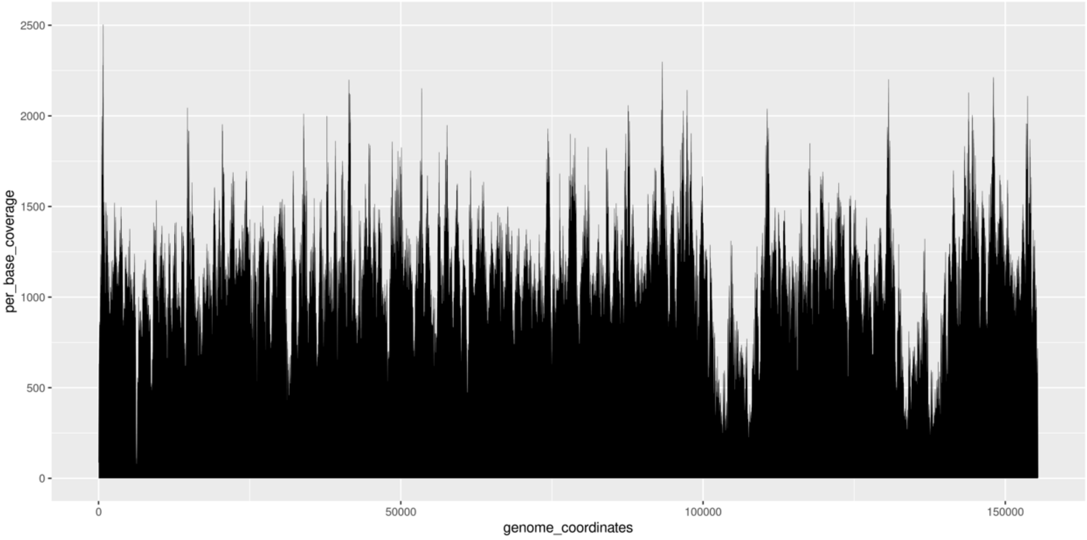

PDS (average read depth/nucleotide: 668.65)

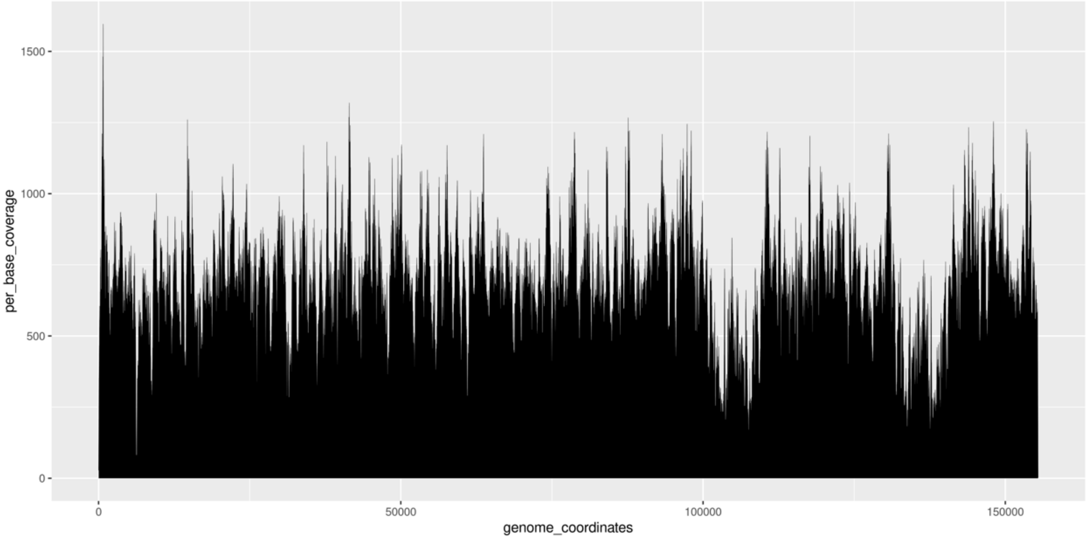

Piennolo giallo (average read depth/nucleotide: 476.94)

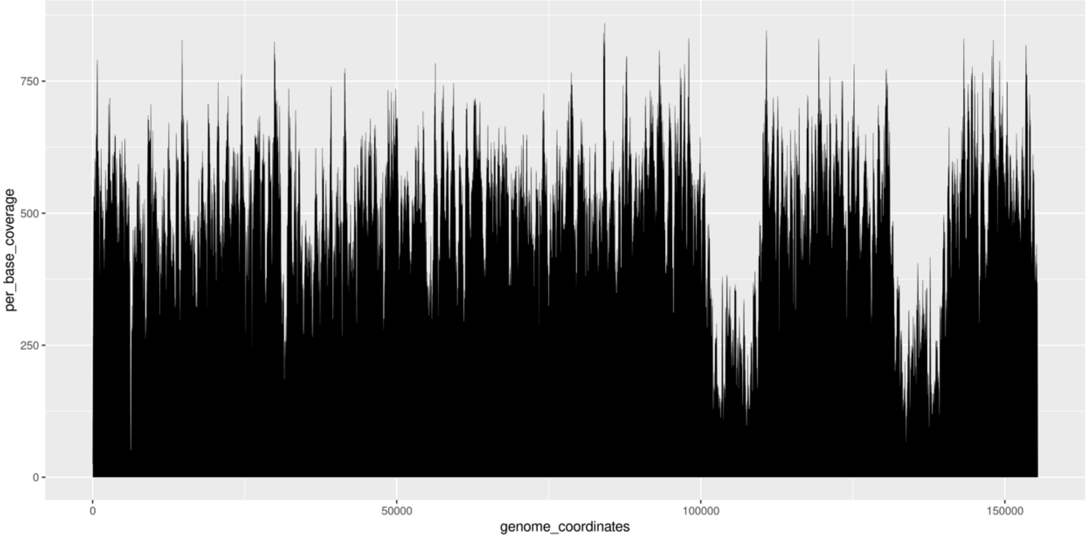

Pollena (average read depth/nucleotide: 1649.77)

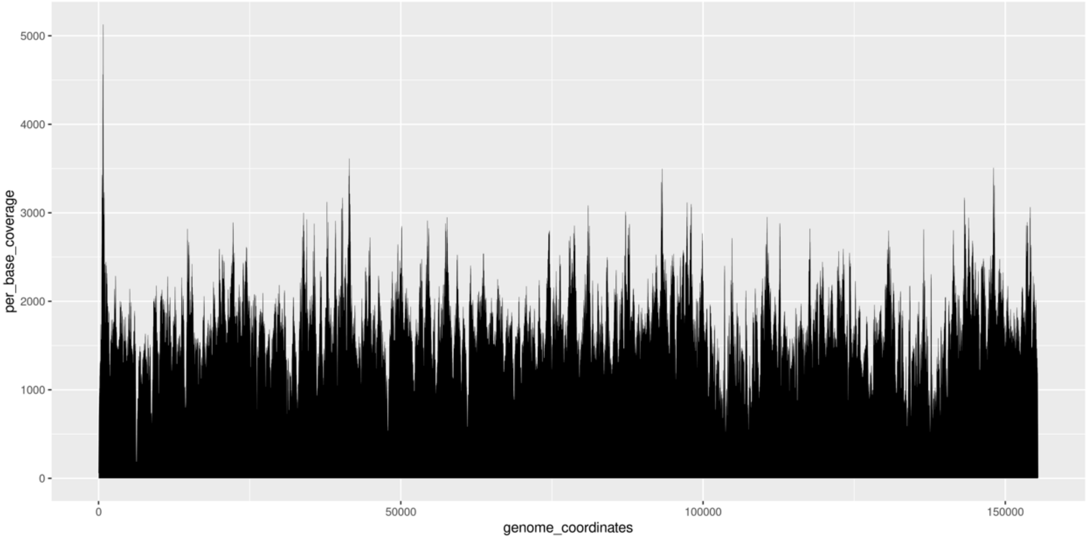

Vesuvio 2001 (average read depth/nucleotide: 1314.46

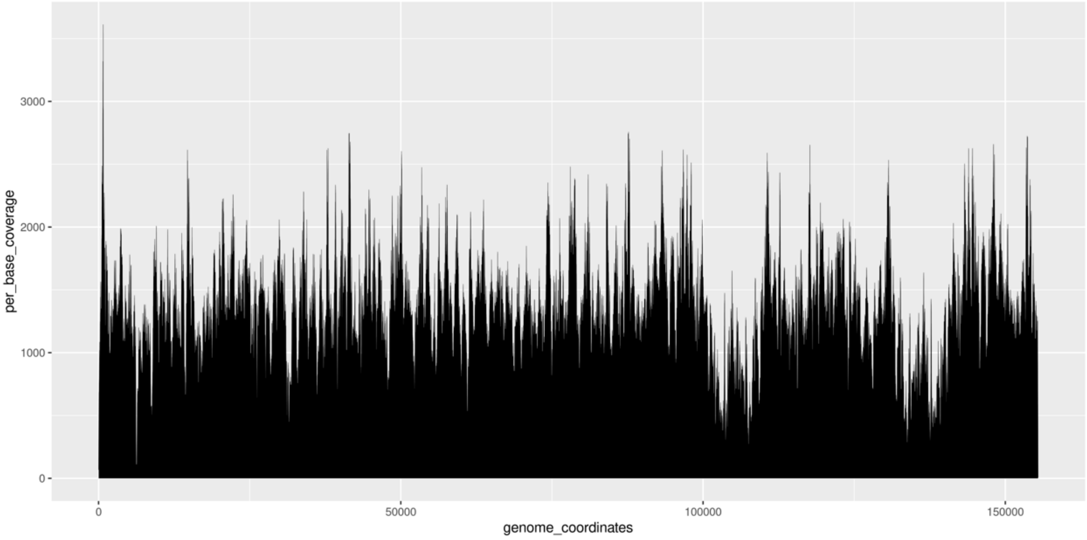

Vesuvio foglia riccia (average read depth/nucleotide: 3147.07)

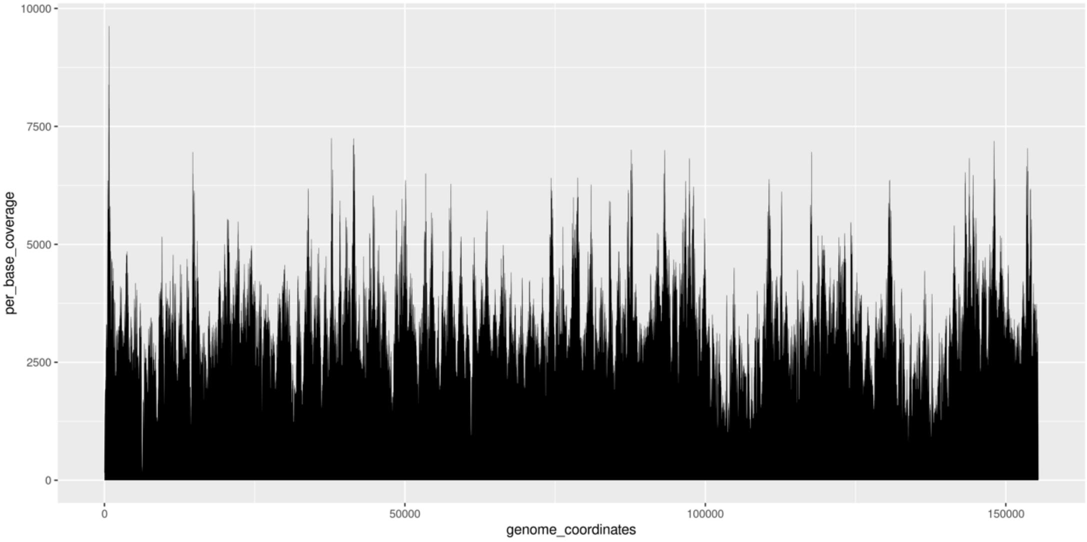

Vesuviano pizzo (average read depth/nucleotide: 600.02)

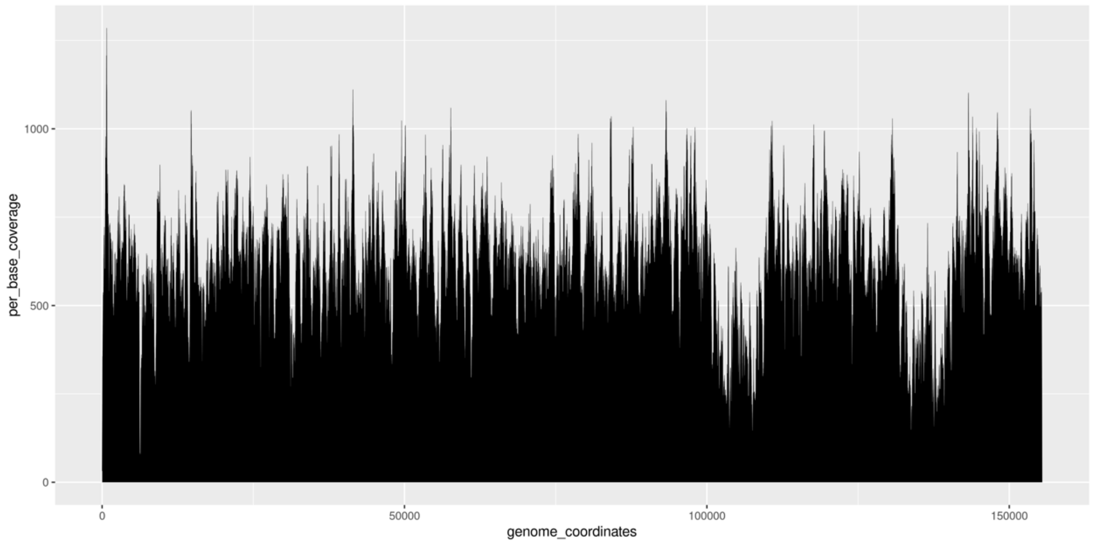

S. neorickii 1 (average read depth/nucleotide: 456.53)

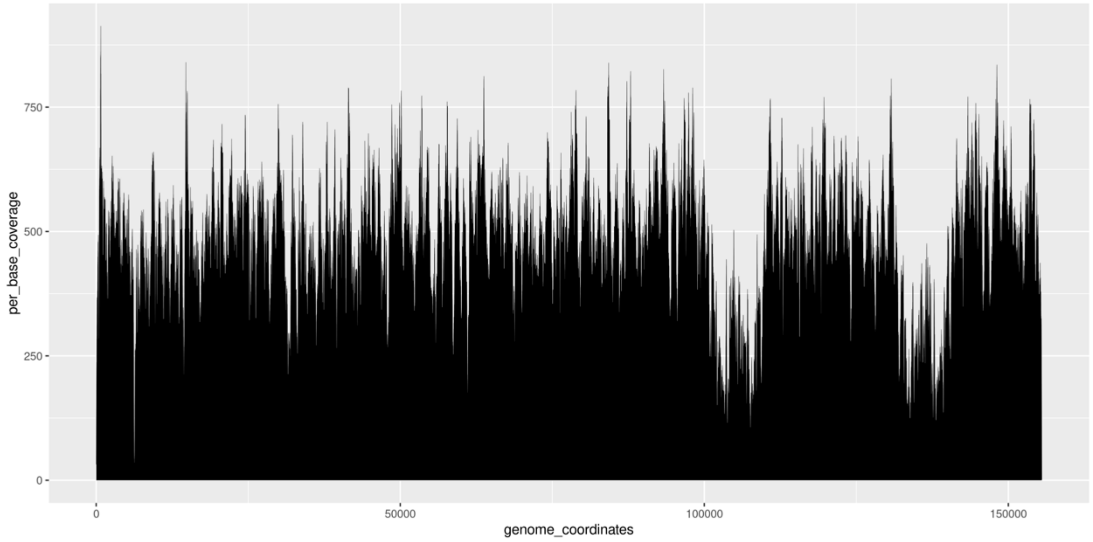

S. pimpinellifolium 1 (average read depth/nucleotide: 975.50)

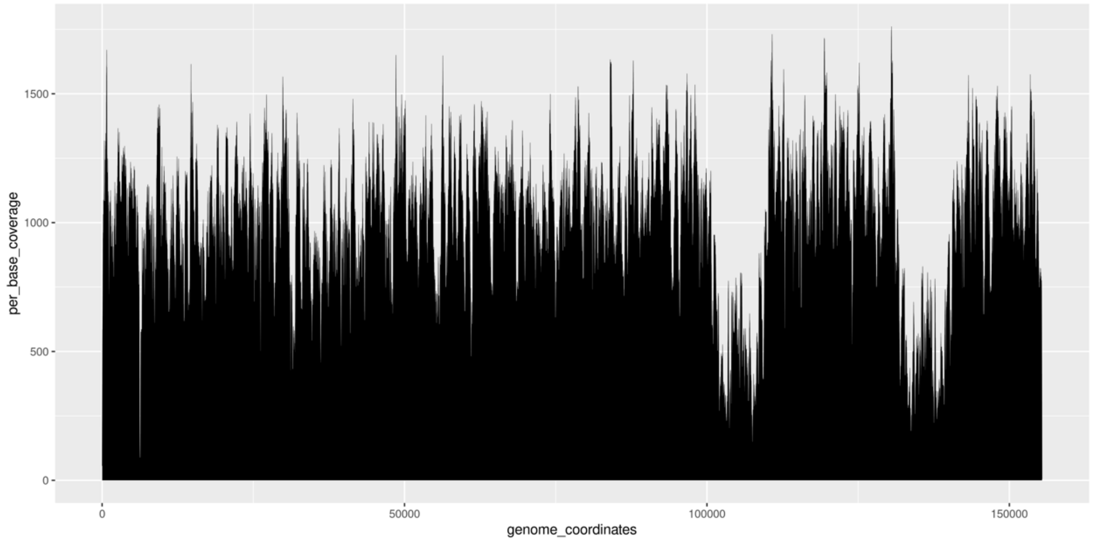

Figure S6. Distribution of *per*-base sequencing depth for each chloroplast genome sequenced in this work. The average coverage *per*-base is also reported.

Table S1. Tomato cpSSR primers developed in this study.

		LOCUS	Repeat	Sequence (5'-3')	Size (bp)	Fluorescent dye	Tm (°C)
LSC	intron	*tRNA-Lys (UUU)*	(T)12	GGTTATCAAATGATACATAGTGC<br>GGAGATGTGTGTAGAAGAAACAG	222	HEX	55
LSC	intron	*tRNA-Gly (UCC)*	(T)13	CTAGAAGGATCATCTACAAAG<br>CGTTAGCTTGGAAGGCTAGG	239	FAM	51
LSC	intergenic	*psbM - tRNA-Asp (GUC)*	(T)16	CGCCTTTCTAAGAGGAGTAGGA<br>CCGATGCATGTAATGGAATCG	314	ATTO550	59
LSC	intergenic	*atpB -rbcL*	(A)13	CATAGATTCATAGAGGAATTCCA<br>GCTTTAGTCTCTGTTTGTGG	389	FAM	55
LSC	intron	*clpP*	(T)17	CCAAACCGCGATGGTATTTTCTT<br>CAATTGGATTGGTAAGATATCTATG	288	ATTO565	58
LSC	intergenic	*rpl14 – rpl16*	(A)10	TGAGCATATCGACGATTGCT<br>CCAAAATGCCTATACGAACTC	262	HEX	55
SSC	intergenic	*ndhF – rpl32*	(A)16-(A)19	TCATTTCTACAGAACAGCCGAT<br>TTCGAGCTAATAGTGTCCTCC	258-261	ATTO550	59
SSC	intergenic	*rpl32 - tRNA-Leu (UAG)*	(A)15	AGACCGCCATGTATATCTTGC<br>CTGAGTCTTTCCTATATTTTCTG	292	ATTO565	58

Table S2. Simple Sequence Repeats (SSRs) in the twenty-one tomato chloroplast genomes using IPA-6 (AM087200) as reference genome. SSRs size, location and distribution among different regions, namely coding, intron, intergenic are reported. The *unit_size/min_repeats* parameters were as follows: 1/8, 2/6, 3/5, 4/5, 5/5, 6/5. SSRs located in IRa were not counted. SSRs were identified using MISA - MIcroSAtellite identification tool (http://pgrc.ipk-gatersleben.de/misa/).

		location	microsatellite	IPA-6	M82	cor	pds	pgl	pol	vpz	ves2001	vfr	cer1	cer2	*S. pimpinellifolium 1*	*S. pimpinellifolium 2*	*S. neorickii 1*	*S. neorickii 2*	*S. habrocaites*	*S. peruvianum*	*S. cheesmaniae*	*S. galapagense*	*S. pennellii*	*S. chilense*
LSC	intergenic	*tRNA-His(GUG) -psbA*	(A)n	13	13	12	12	12	12	12	12	12	12	12	12	12	15	15	18	12	13	13	14	13
LSC	coding	*matK*	(T)n	8	8	8	8	8	8	8	8	8	8	8	8	8	8	8	8	8	8	8	8	8
LSC	coding	*matK*	(T)n	9	9	9	9	9	9	9	9	9	9	9	9	9	9	9	9	9	9	9	9	9
LSC	intron	*tRNA-Lys (UUU)*	(T)n	12	12	12	12	12	12	12	12	12	11	12	11	11	13	13	11	11	12	12	10	11
LSC	intergenic	*tRNA-Lys (UUU) – rps16*	(A)n	11	11	11	11	11	11	11	11	11	11	11	11	11	8	8	11	10	10	10	9	10
LSC	intergenic	*tRNA-Lys (UUU) – rps16*	(AT)n	4	4	4	4	4	4	4	4	4	4	4	4	4	4	4	4	7	4	4	4	7
LSC	intergenic	*rps16 - tRNA-Gln (UUG)*	(T)n	7	7	7	7	7	7	7	7	7	7	7	7	7	7	7	7	7	7	8	7	7
LSC	intergenic	*rps16 - tRNA-Gln (UUG)*	(T)n	16	16	16	16	16	16	16	16	16	16	16	15	15	11	11	15	12	16	16	-	12
LSC	intergenic	*rps16 - tRNA-Gln (UUG)*	(T)n	9	9	9	9	9	9	9	9	9	9	9	9	9	13	13	16	13	9	9	13	13
LSC	intergenic	*rps16 - tRNA-Gln (UUG)*	(A)n	11	11	11	11	11	11	11	11	11	12	11	12	12	13	13	14	11	11	11	14	11
LSC	intergenic	*psbK - psbI*	(T)n	11	11	11	11	11	11	11	11	11	11	11	11	11	12	12	12	11	10	10	11	11
LSC	intergenic	*psbI – tRNA-Ser (GCU)*	(A)n	11	11	11	11	11	11	11	11	11	11	11	11	11	10	10	9	10	10	10	10	10
LSC	intergenic	*tRNA-Ser (GCU) - tRNA-Gly (UCC)*	(T)n	7	7	7	7	7	7	7	7	7	7	7	7	7	8	8	7	8	7	7	7	8
LSC	intergenic	*tRNA-Ser (GCU) - tRNA-Gly (UCC)*	(A)n	9	9	9	9	9	9	9	9	9	9	9	9	9	9	9	9	9	9	9	9	9
LSC	intergenic	*tRNA-Ser (GCU) - tRNA-Gly (UCC)*	(A)n	8	8	8	8	8	8	8	8	8	9	8	9	9	8	8	8	8	8	8	8	8
LSC	intron	*tRNA-Gly (UCC)*	(T)n	13	13	13	13	13	13	13	13	13	12	13	13	13	13	14	11	10	13	13	13	10
LSC	intergenic	*tRNA-Gly (UCC) - tRNA-Arg (UCU)*	(T)n	12	12	12	12	12	12	12	12	12	12	12	12	12	11	11	12	13	12	12	11	13
LSC	intergenic	*tRNA-Gly (UCC) - tRNA-Arg (UCU)*	(A)n	7	7	7	7	7	7	7	7	7	7	7	7	7	8	8	7	8	7	7	8	8
LSC	intergenic	*tRNA-Arg (UCU) – atpA*	(T)n	9	9	9	9	9	9	9	9	9	9	9	9	9	10	10	9	10	9	9	9	10
LSC	intergenic	*atpA -atpF*	(A)n	8	8	8	8	8	8	8	8	8	8	8	8	8	8	8	8	8	8	8	8	8
LSC	intron	*atpF*	(T)n	9	9	9	9	9	9	9	9	9	9	9	9	9	9	9	9	9	9	9	9	9
LSC	coding	*atpF*	(A)n	8	8	8	8	8	8	8	8	8	8	8	8	8	8	8	8	8	8	8	8	8
LSC	intergenic	*atpH – atpI*	(A)n	8	8	8	8	8	8	8	8	8	8	8	8	8	8	8	8	8	8	8	8	8
LSC	intergenic	*atpH – atpI*	(T)n	8	8	8	8	8	8	8	8	8	9	8	9	9	8	8	8	8	8	8	8	8
LSC	intergenic	*rps2 - rpoC2*	(A)n	11	11	11	11	11	11	11	11	11	11	11	10	11	11	11	11	11	11	11	11	11
LSC	coding	*rpoC2*	(T)n	8	8	8	8	8	8	8	8	8	8	8	8	8	8	8	8	8	8	8	8	8
LSC	coding	*rpoC2*	(C)n	8	8	8	8	8	8	8	8	8	8	8	8	8	8	8	8	8	8	8	8	8
LSC	coding	*rpoC2*	(T)n	10	10	10	10	10	10	10	10	10	10	10	10	10	10	10	10	10	10	10	10	10
LSC	coding	*rpoC2*	(A)n	8	8	8	8	8	8	8	8	8	8	8	8	8	8	8	8	8	8	8	8	8
LSC	coding	*rpoC1*	(T)n	11	11	11	11	11	11	11	11	11	11	11	11	11	11	11	11	11	11	11	11	11
LSC	coding	*rpoC1*	(A)n	8	8	8	8	8	8	8	8	8	8	8	8	8	8	8	8	8	8	8	8	8
LSC	coding	*rpoB*	(T)n	8	8	8	8	8	8	8	8	8	8	8	8	8	8	8	8	8	8	8	8	8
LSC	intergenic	*tRNA-Cys (GCA) - petN*	(TA)n	3	3	3	3	3	3	3	3	3	3	3	3	3	6	6	3	3	3	3	3	3
LSC	intergenic	*petN – psbM*	(A)n	11	11	11	11	11	11	11	11	11	11	11	11	11	11	11	11	11	11	11	11	11
LSC	intergenic	*psbM - tRNA-Asp (GUC)*	(T)n	16	16	16	16	16	16	16	16	16	16	16	16	15	10	10	15	11	16	16	13	11
LSC	intergenic	*tRNA-Glu (UUC) - tRNA-Thr (GGU)*	(T)n	8	8	8	8	8	8	8	8	8	9	8	9	9	9	9	9	9	9	9	9	9
LSC	intergenic	*tRNA-Thr (GGU) – psbD*	(T)n	8	8	8	8	8	8	8	8	8	8	8	8	8	8	8	8	8	8	8	8	8
LSC	intergenic	*psbC - tRNA-Ser (UGA)*	(T)n	12	12	12	12	12	12	12	12	12	12	12	12	12	10	10	12	10	13	12	10	10
LSC	intron	*ycf3*	(T)n	8	8	8	8	8	8	8	8	8	8	8	8	8	8	8	8	8	8	8	8	8
LSC	intron	*ycf3*	(A)n	11	11	11	11	11	11	11	11	11	11	11	11	11	9	9	11	9	11	11	9	9
LSC	intergenic	*ycf3 - tRNA-Ser (GGA)*	(T)n	8	8	8	8	8	8	8	8	8	8	8	8	8	8	8	8	8	8	8	8	8
LSC	intergenic	*ycf3 - tRNA-Ser (GGA)*	(A)n	8	8	8	8	8	8	8	8	8	7	8	7	7	7	7	7	7	7	7	7	7
LSC	intergenic	*rps4 - tRNA-Thr (UGU)*	(T)n	10	10	10	10	10	10	10	10	10	9	10	9	9	6	6	10	6	9	9	6	6
LSC	intergenic	*rps4 - tRNA-Thr (UGU)*	(TA)n	6	6	6	6	6	6	6	6	6	6	6	6	6	6	6	6	6	6	6	6	6
LSC	intergenic	*rps4 - tRNA-Thr (UGU)*	(A)n	10	10	10	10	10	10	10	10	10	11	10	11	11	11	11	11	10	12	12	13	10
LSC	intergenic	*tRNA-Thr (UGU) - tRNA-Leu (UAA)*	(T)n	11	11	11	11	11	11	11	11	11	9	11	9	9	5	5	12	5	10	10	5	5
LSC	intergenic	*tRNA-Leu (UAA) - tRNA-Phe (GAA)*	(T)n	8	8	8	8	8	8	8	8	8	8	8	8	8	8	8	8	8	8	8	8	8
LSC	coding	*ndhC*	(A)n	8	8	8	8	8	8	8	8	8	8	8	8	8	8	8	8	8	8	8	8	8
LSC	intergenic	*ndhC – tRNA-Val (UAC)*	(T)n	10	10	9	9	9	9	9	9	9	10	10	10	10	9	9	10	9	10	10	10	9
LSC	intron	*tRNA-Val (UAC)*	(T)n	8	8	8	8	8	8	8	8	8	8	8	8	8	8	8	8	8	8	8	8	8
LSC	intron	*tRNA-Val (UAC)*	(T)n	8	8	8	8	8	8	8	8	8	8	8	8	8	8	8	8	8	8	8	8	8
LSC	intergenic	*tRNA-Met (CAU) – atpE*	(T)n	12	12	12	12	12	12	12	12	12	13	12	13	13	11	11	14	11	13	13	10	11
LSC	coding	*atpB*	(T)n	10	10	10	10	10	10	10	10	10	10	10	10	10	10	10	9	11	10	10	10	10
LSC	intergenic	*atpB -rbcL*	(A)n	8	8	8	8	8	8	8	8	8	8	8	8	8	4	4	8	4	8	8	4	4
LSC	intergenic	*atpB -rbcL*	(A)n	13	13	13	13	13	13	13	13	13	12	12	12	12	12	12	12	12	12	12	12	12
LSC	intergenic	*rbcL – accD*	(T)n	8	8	8	8	8	8	8	8	8	8	8	8	8	8	8	8	8	8	8	8	8
LSC	intergenic	*accD – psaI*	(T)n	8	8	8	8	8	8	8	8	8	8	8	8	8	8	8	8	8	8	8	8	8
LSC	intergenic	*psaI - ycf4*	(T)n	10	10	10	10	10	10	10	10	10	11	10	11	11	10	10	11	10	11	11	10	10
LSC	coding	*ycf4*	(T)n	8	8	8	8	8	8	8	8	8	8	8	8	8	8	8	8	8	8	8	8	8
LSC	intergenic	*ycf4 – ycf10*	(T)n	8	8	8	8	8	8	8	8	8	8	8	8	8	8	8	8	8	8	8	8	8
LSC	intergenic	*ycf4 – ycf10*	(T)n	10	10	10	10	10	10	10	10	10	10	10	10	10	12	12	10	12	10	10	11	12
LSC	intergenic	*petA – psbJ*	(T)n	9	9	9	9	9	9	9	9	9	9	9	9	9	9	9	9	9	9	9	9	9
LSC	coding	*psbF*	(A)n	8	8	8	8	8	8	8	8	8	8	8	8	8	8	8	8	8	8	8	8	8
LSC	intergenic	*psbE – petL*	(T)n	9	9	8	8	8	8	8	8	8	8	8	8	8	8	8	8	8	8	8	8	8
LSC	intergenic	*psbE – petL*	(A)n	8	8	8	8	8	8	8	8	8	8	8	8	8	8	8	8	8	8	8	8	8
LSC	intergenic	*petL – petG*	(T)n	10	10	10	10	10	10	10	10	10	9	10	9	9	9	9	10	9	9	9	9	9
LSC	intergenic	*petL – petG*	(A)n	8	8	8	8	8	8	8	8	8	10	8	10	10	9	9	9	9	9	9	9	9
LSC	intergenic	*tRNA-Pro (UGG) – psaJ*	(T)n	10	10	10	10	10	10	10	10	10	10	10	10	10	9	9	10	9	10	10	9	9
LSC	intergenic	*rpl20 – rps12*	(T)n	9	9	9	9	9	9	9	9	9	8	9	8	8	9	9	9	9	8	8	9	9
LSC	intergenic	*rpl20 – rps12*	(T)n	10	10	10	10	10	10	10	10	10	10	10	10	10	10	10	10	10	10	10	10	10
LSC	intron	*clpP*	(T)n	8	8	8	8	8	8	8	8	8	8	8	8	8	8	8	8	8	8	8	8	8
LSC	intron	*clpP*	(T)n	9	9	9	9	9	9	9	9	9	9	9	9	9	9	9	10	9	9	9	9	9
LSC	intron	*clpP*	(A)n	11	11	11	11	11	11	11	11	11	11	11	11	11	11	11	10	10	11	11	11	10
LSC	intron	*clpP*	(T)n	8	8	8	8	8	8	8	8	8	6	8	6	6	6	6	6	6	6	6	6	6
LSC	intron	*clpP*	(T)n	17	17	17	17	17	17	17	17	17	17	17	16	17	13	13	17	16	17	17	14	16
LSC	intergenic	*clpP -psbB*	(T)n	8	8	8	8	8	8	8	8	8	8	8	8	8	8	8	8	8	8	8	8	8
LSC	intergenic	*psbB -psbT*	(A)n	8	8	8	8	8	8	8	8	8	8	8	8	8	9	9	8	9	8	8	9	9
LSC	intergenic	*psbT – psbN*	(T)n	9	9	9	9	9	9	9	9	9	8	9	8	8	9	9	9	9	9	9	8	9
LSC	coding	*rpoA*	(T)n	10	10	10	10	10	10	10	10	10	10	10	10	10	10	10	10	10	10	10	10	10
LSC	intergenic	*rpl14 – rpl16*	(A)n	10	10	10	10	10	10	10	10	10	9	10	9	9	9	9	9	9	9	9	9	9
LSC	intergenic	*rpl14 – rpl16*	(A)n	8	8	8	8	8	8	8	8	8	8	8	8	8	8	8	8	8	8	8	8	8
LSC	intron	*rpl16*	(A)n	8	8	8	8	8	8	8	8	8	8	8	8	8	8	8	8	8	8	8	8	8

LSC	intron	*rpl16*	(T)n	7	7	7	7	7	7	7	7	7	7	7	7	7	7	7	7	8	7	7	7	8
LSC	intergenic	*rpl16 – rps3*	(T)n	9	9	9	9	9	9	9	9	9	9	9	9	9	9	9	9	9	9	9	9	9
LSC	coding	*rpl22*	(T)n	9	9	9	9	9	9	9	9	9	9	9	9	9	9	9	9	9	9	9	9	9
LSC	coding	*rpl22*	(T)n	8	8	8	8	8	8	9	8	8	8	8	8	8	8	8	8	8	8	8	8	8
Irb	coding	*rps19*	(T)n	8	8	8	8	8	8	9	8	8	8	8	8	8	8	8	8	8	8	8	8	8
Irb	coding	*rps19*	(T)n	9	9	9	9	9	9	8	9	9	9	9	9	9	9	9	9	9	9	9	9	9
Irb	intergenic	*rps19 – rpl2*	(T)n	10	10	9	9	9	9	9	9	9	9	10	10	10	10	11	11	10	10	10	10	11	10
Irb	coding	*ycf2*	(A)n	9	9	9	9	9	9	9	9	9	9	9	9	9	9	9	9	9	9	9	9	9
Irb	intergenic	*ndhB – rps7*	(A)n	10	10	10	10	10	10	10	10	10	10	10	10	10	9	9	10	9	10	10	9	9
Irb	coding	*rps7*	(T)n	9	9	9	9	9	9	9	9	9	9	9	9	9	9	9	9	9	9	9	9	9
Irb	intergenic	*rps12 - tRNA-Val (GAC)*	(T)n	8	8	8	8	8	8	8	8	8	8	8	8	8	8	8	8	8	8	8	8	8
Irb	intergenic	*rrn16 - tRNA-Ile (GAU)*	(A)n	8	8	8	8	8	8	8	8	8	8	8	8	8	8	8	8	8	8	8	8	8
Irb	intergenic	*rrn5 - tRNA-Arg (ACG)*	(A)n	10	10	10	10	10	10	10	10	10	11	10	10	10	10	10	10	10	10	10	10	10
Irb	coding	*ORF380 truncated ycf1*	(A)n	-	-	-	-	-	-	-	-	-	-	-	-	-	-	-	-	8	-	-	-	-
Irb	coding	*ORF380 truncated ycf1*	(A)n	8	8	8	8	8	8	8	8	8	8	8	8	8	8	8	8	8	8	8	8	8
Irb	coding	*ORF380 truncated ycf1*	(A)n	-	-	-	-	-	-	-	-	-	-	-	-	-	-	-	-	-	8	-	-	-
SSC	intergenic	*ndhF – rpl32*	(A)n	19	19	16	16	15	16	16	16	16	19	19	16	16	6	6	13	11	15	14	12	10
SSC	intergenic	*rpl32 - tRNA-Leu (UAG)*	(A)n	8	8	8	8	8	8	8	8	8	7	8	7	7	7	7	7	7	7	7	7	7
SSC	intergenic	*rpl32 - tRNA-Leu (UAG)*	(A)n	15	15	15	15	15	15	15	15	15	17	17	18	18	16	13	15	13	19	19	13	14
SSC	intergenic	*rpl32 - tRNA-Leu (UAG)*	(A)n	9	8	8	8	8	8	8	8	8	8	8	8	8	8	8	8	8	8	8	8	8
SSC	coding	*ccsA*	(A)n	8	8	8	8	8	8	8	8	8	8	8	8	8	8	8	8	8	8	8	8	8
SSC	intergenic	*ccsA -ndhD*	(A)n	7	7	7	7	7	7	7	7	7	7	7	7	7	7	7	7	8	7	7	7	9
SSC	coding	*ndhD*	(A)n	8	8	8	8	8	8	8	8	8	8	8	8	8	8	8	8	8	8	8	8	8
SSC	coding	*ndhD*	(T)n	8	8	8	8	8	8	8	8	8	8	8	8	8	8	8	8	8	8	8	8	8
SSC	intron	*ndhA*	(A)n	8	8	8	8	8	8	8	8	8	8	8	8	8	9	9	8	9	8	8	9	9
SSC	coding	*ndhA*	(A)n	9	9	9	9	9	9	9	9	9	9	9	9	9	9	9	9	9	9	9	9	9
SSC	coding	*rps15*	(T)n	9	9	9	9	9	9	9	9	8	9	9	9	9	9	9	9	9	9	9	9	9
SSC	intergenic	*rps15 – ycf1*	(T)n	8	8	8	8	8	8	8	8	8	8	8	8	8	8	8	8	8	8	8	8	8
SSC	coding	*ycf1*	(T)n	10	10	10	10	10	10	10	10	10	10	10	10	10	10	10	10	10	10	10	10	10
SSC	coding	*ycf1*	(T)n	8	8	8	8	8	8	8	8	8	8	8	8	8	8	8	8	8	8	8	7	8
SSC	coding	*ycf1*	(T)n	8	8	8	8	8	8	8	8	8	8	8	8	8	8	8	8	8	8	8	8	8
SSC	coding	*ycf1*	(T)n	9	9	9	9	9	9	9	9	9	9	9	9	9	9	9	9	9	9	9	9	9

Table S3. Tandem Repeats (TRs) in the twenty-one tomato chloroplast genomes using IPA-6 (AM087200) as reference genome. TRs copy number and location are reported. TRs were identified using the tool available at https://tandem.bu.edu/trf/trf.basic.submit.html.

Sequence	Period Size	Copy Number																					location		
		IPA-6	M82	cor	pds	pgl	pol	vpz	ves2001	vfr	cer1	cer2	*S. pimpinellifolium 1*	*S. pimpinellifolium 2*	*S. neorickii 1*	*S. neorickii 2*	*S. habrocaites*	*S. peruvianum*	*S. cheesmaniae*	*S. galapagense*	*S. pennellii*	*S. chilense*			
GTTCAATGATATTATTATT	19	-	-	-	-	-	-	-	-	-	-	-	-	-	-	-	-	2.0	-	-	-	2.0	LSC	*tRNA-His (GUG) – psbA*	intergenic
ATCATGAATAAAT	13	2.0	2.0	2.0	2.0	2.0	2.0	2.0	2.0	2.0	2.0	2.0	2.0	2.0	-	-	2.0	-	2.0	2.0	-	-	LSC	*tRNA-His (GUG) – psbA*	intergenic
TATTTCCTTAATT	13	2.0	2.0	2.0	2.0	2.0	2.0	2.0	2.0	2.0	2.0	2.0	2.0	2.0	2.0	2.0	2.0	2.0	2.0	2.0	2.0	2.0	LSC	*rps16*	genic
TAATACTATAGAT	13	2.0	2.0	2.0	2.0	2.0	2.0	2.0	2.0	2.0	2.0	2.0	2.0	2.0	2.0	2.0	2.0	2.0	2.0	2.0	2.0	2.0	LSC	*rps16 - tRNA-Gln (UUG)*	intergenic
ACTTACTTTCCTTTTCCTTATA	22	2.0	2.0	-	-	-	-	-	-	-	2.0	2.0	-	2.0	3.0	3.0	2.0	2.0	2.0	2.0	2.0	2.0	LSC	*tRNA-Gln (UUG) – psbK*	intergenic
GGTTTAAGTCTGA	13	2.0	2.0	2.0	2.0	2.0	2.0	2.0	2.0	2.0	2.0	2.0	2.0	2.0	2.0	2.0	2.0	2.0	2.0	2.0	2.0	2.0	LSC	*rps4*	genic
ACGTAATACATATTAGAAAG	20	-	-	-	-	-	-	-	-	-	-	-	-	-	2.0	2.0	-	2.0	-	-	2.0	2.0	LSC	*tRNA-Phe (GAA) – ndhJ*	intergenic
AGTTTAATAGTTTACCC	17	-	-	-	-	-	-	-	-	-	-	-	-	-	-	-	-	-	-	-	2.1	-	LSC	*PsbE – petL*	intergenic
TTTTTTCATTCTAGTTA	17	-	-	-	-	-	-	-	-	-	-	-	-	-	2.1	2.1	-	2.1	-	-	-	2.1	LSC	*psaJ – rpl33*	intergenic
GAATCTAGATTTGAGT	16	2.0	2.0	2.0	2.0	2.0	2.0	2.0	2.0	2.0	2.0	2.0	2.0	2.0	2.0	2.0	2.0	2.0	2.0	2.0	2.0	2.0	LSC	*rps18 – rpl20*	intergenic
CATAGATATCTTA	13	2.1	2.1	2.1	2.1	2.1	2.1	2.1	2.1	2.1	2.1	2.1	2.1	2.1	2.1	2.1	2.1	2.1	2.1	2.1	2.1	2.1	LSC	*clpP*	intronic
AGAATTAAGAATTAGAA	17	2.1	2.1	2.1	2.1	2.1	2.1	2.1	2.1	2.1	2.1	2.1	2.1	2.1	-	-	2.1	-	2.1	2.1	-	-	LSC	*rpl16 -rps3*	intergenic
AATCAATGCAATTTAGGAGG	20	3.4	3.4	3.4	3.4	3.4	3.4	3.4	3.4	3.4	3.4	3.4	3.4	3.4	3.4	3.4	3.4	3.4	3.4	3.4	3.4	3.4	Irb	*tRNA-Ile – ycf2*	intergenic
ACTTAGTGAGTCCTTTCTTC	20	2.0	2.0	2.0	2.0	2.0	2.0	2.0	2.0	2.0	2.0	2.0	2.0	2.0	2.0	2.0	2.0	2.0	2.0	2.0	2.0	2.0	Irb	*rps12 - tRNA-Val (GAC)*	intergenic
GTCATTAATATCTTTG	16	-	-	-	-	-	-	-	-	-	-	-	-	-	-	-	-	2.0	-	-	-	2.0	Irb	*tRNA-Arg (ACG) - tRNA-Asn (GUU)*	intergenic
AGAAGAACGTATATAAAA	18	-	-	-	-	-	-	-	-	-	-	-	-	-	2.1	2.1	-	-	-	-	-	-	SSC	*rpl32 - tRNA-Leu (UAG)*	intergenic
TTGTTTTAGATCCGAACTAAGCCTTC	26	-	-	-	-	-	-	-	-	-	-	-	-	-	-	-	-	-	2.0	2.0	-	-	SSC	*ndhG – ndhI*	intergenic
